# Odour-mediated oviposition site selection in *Aedes aegypti* depends on aquatic stage and density

**DOI:** 10.1186/s13071-023-05867-1

**Published:** 2023-08-04

**Authors:** Zaid Khan, Björn Bohman, Rickard Ignell, Sharon Rose Hill

**Affiliations:** Disease Vector Group, Department of Plant Protection Biology, Swedish University of Agricultural Sciences, Box 190, 234 22 Lomma, Sweden

**Keywords:** Mosquito behaviour, Immature stages, Volatile organic compounds, Synthetic odour blends

## Abstract

**Background:**

Olfaction plays an important role in the selection and assessment of oviposition sites by mosquitoes. Volatile organic compounds (VOCs) associated with potential breeding sites affect the behaviour of gravid mosquitoes, with VOCs from aquatic stages of conspecific mosquitoes influencing and regulating oviposition. The purpose of this study was to conduct a systematic analysis of the behavioural response of gravid *Aedes aegypti* to conspecific aquatic stage-conditioned water, to identify the associated bioactive VOCs and to determine how blends of these VOCs regulate oviposition site selection and stimulate egg-laying.

**Methods:**

Using a multi-choice olfactory oviposition assay, controlling for other sensory modalities, the responses of individual females to water conditioned with different densities of conspecific aquatic stages were assessed. The conditioned water samples from the most preferred density of each aquatic stage were subsequently compared to each other using the same oviposition assay and analysed using an analysis of variance (ANOVA) followed by a Tukey post-hoc test. Using combined gas chromatography and electroantennographic detection or mass spectrometry, bioactive VOCs from the preferred density of each aquatic stage were identified. Synthetic blends were prepared based on the identified ratios of bioactive VOCs in the aquatic stages, and then tested to determine the oviposition choice of *Ae. aegypti* in a dose-dependent manner, against a solvent control, using a dual-choice assay. This dataset was analysed using nominal logistic regression followed by an odds ratio comparison.

**Results:**

Gravid *Ae. aegypti* responded stage- and density-dependently to water conditioned with eggs, second- and fourth-instar larvae, and pupal exuviae, but not to water conditioned with pupae alone. Multi-choice assays demonstrated that gravid mosquitoes preferred to oviposit in water conditioned with fourth-instar larvae, over the other aquatic stage-conditioned water. Gravid *Ae. aegypti* were attracted, and generally stimulated, to oviposit in a dose-dependent manner to the individual identified synthetic odour blends for the different aquatic stages.

**Conclusions:**

Intraspecific VOCs regulate oviposition site selection in *Ae. aegypti* in a stage- and density-dependent manner. We discuss the need for further studies to evaluate the identified synthetic blends to modulate the odour-mediated oviposition of *Ae. aegypti* under field conditions.

**Graphical Abstract:**

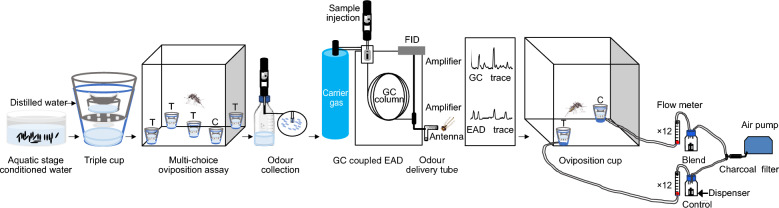

**Supplementary Information:**

The online version contains supplementary material available at 10.1186/s13071-023-05867-1.

## Background

For mosquitoes, oviposition site selection is essential, as this decision directly regulates the growth, development and survival of the next generation, as well as population dynamics [1–5]. The aquatic stages of mosquitoes are limited in their movement, and thus the fate of the offspring is largely dependent on the maternal selection of oviposition sites [2, 6, 7]. While seeking oviposition sites, gravid mosquitoes must search for, and distinguish between, potential oviposition sites over multiple spatial scales to ensure the availability of nutrients for larval development and survival, and to reduce competition and offspring mortality [2, 8–10]. For this purpose, mosquitoes rely predominantly on olfactory cues emanating from potential oviposition sites and their surroundings [6, 9, 11]. Emanates from conspecific immature stages associated with breeding sites can act as reliable signals for females to assess the quality of an oviposition site, in terms of overcrowding and competition from con- and heterospecific aquatic stages [11]. An increased understanding of the signals regulating conspecific oviposition site selection may lead to the development of species- and/or genera-specific attractants for vector control.

Oviposition site selection by gravid mosquitoes can be modulated by cues associated with the aquatic stages, in a species-, stage- and density-dependent manner [11]. Gravid mosquitoes generally avoid ovipositing in breeding sites in which the risk of con- and heterospecific competition and cannibalism/predation is high [12–19]. The manner by which individual species assess oviposition sites differs in accordance with species-specific breeding site requirements [2, 6, 11, 20]. This is, therefore, dependent on the ability of gravid females to evaluate cues emanating from distinct conspecific aquatic stages, as these provide reliable signals of breeding site conditions [11, 12, 15, 18, 19]. Limitation in nutrient resources, regulated by, e.g., competition and the dynamic nature of mosquito-associated microbial communities, differentially affects oviposition site choice in a taxon-dependent manner [5, 6, 19, 21, 22]. In addition, the persistence of individual breeding sites affects mosquitoes species-specifically, often depending on the drought tolerance of the aquatic stages, with conspecific stage-associated cues providing reliable temporal information concerning, e.g., ephemeral and cyclically flooded sites [2, 6, 21]. For example, gravid yellow fever and Asian tiger mosquitoes *Aedes aegypti* and *Aedes albopictus*, respectively, preferentially select breeding sites that contain or have previously contained, late aquatic stages, which is believed to indicate the availability of larval food resources [20, 22]. The density of the conspecific aquatic stages in the breeding sites also modulates mosquito oviposition site selection, as overcrowding leads to competition while low conspecific densities increase the risk of predation, with optimal densities being species-dependent [12, 15, 17, 18, 23–25]. While a limited number of behaviourally active volatile organic compounds (VOCs) have been identified associated with conspecific aquatic stages, there is a further need for a systematic cross-disciplinary chemical ecological analysis to identify intraspecific signals regulating oviposition site selection and egg laying in mosquitoes [11].

This study aimed at exploring the stage- and density-dependent behavioural response of the yellow fever mosquito *Ae. aegypti* during oviposition site selection and egg-laying, as well as identifying the natural bioactive VOCs associated with water conditioned with aquatic stages, regulating this choice. Synthetic blends of these bioactive VOCs were evaluated for their ability to manipulate the oviposition response of gravid *Ae. aegypti*. Understanding oviposition in mosquitoes, and the bioactive VOCs involved, will assist in enhancing existing vector surveillance and control programmes by targeting gravid mosquitoes to reduce vector populations and the burden of disease transmission.

## Methods

### Mosquito rearing

*Aedes aegypti* (Rockefeller) eggs, laid on filter paper (90 mm diameter; Ahlstrom, Munksjö, Finland), were placed in 3-l plastic rearing trays (L: 24.5 × W: 18.5 × H: 7.5 cm; Emballator Lagan AB, Ljungby, Sweden) filled with 1 l distilled water. Larvae were fed daily with TetraMin^®^ fish food (Tetra GmbH, Melle, Germany). The pupae were collected in 30-ml containers (Essentra Components, Malmö, Sweden) kept in a BugDorm-1 cage (L: 30 × W: 30 × H: 30 cm; MegaView Science, Taichung, Taiwan) for adult emergence. Adults were maintained at 27 ± 1 °C, 65 ± 5% relative humidity, and at a 12:12 h light–dark cycle, with ad libitum access to a 10% sucrose solution. For colony maintenance and experiments, adults 5–7 days post-emergence (dpe) were provided with sheep blood (Håtunalab, Bro, Sweden) for 2 h using a collagen membrane through a Hemotek membrane feeding system (Hemotek Ltd., Blackburn, UK) at 37 °C. Fully engorged females were used in the oviposition experiments, five days after blood meal ingestion (10–12 dpe).

### Conditioning water with aquatic stages of *Aedes aegypti*

Water was conditioned with eggs, second-instar larvae, fourth-instar larvae, pupae or pupal casings of *Ae. aegypti*. To produce the egg-conditioned water (ECW), filter papers containing c. 250, 500, 1000 and 2000 eggs were collected and incubated at −20 °C for 30 min to prevent hatching. This treatment is unlikely to have damaged the chorion or outer egg casing [26], and thus unlikely to affect the VOCs released. Individual filter papers, with or without eggs, were then placed in rearing trays containing 1 l distilled water for 22 ± 2 h. To obtain the larvae-conditioned water (LCW), first-instar larvae were transferred to rearing trays (see above) containing one of four densities (c. 50, 150, 300 and 600 larvae l^−1^), and reared to second instar or fourth instar without changing the water prior to subsequent assays. Throughout its development, each larva was fed c. 10 mg of TetraMin fish food, with first- to early third-instar larvae provided with 0.6 mg of food larva per day, and late third- and fourth-instar larvae provided with 2 mg of food larva per day. As a control, distilled water was treated with equivalent amounts of fish food and under the same conditions, with food particles sieved out daily prior to the next allotment of fish food to reflect the consumption of food by the larvae. To generate the water conditioned with pupae and their exuviae, two groups of pupae were collected. The pupae, at densities of either c. 50, 100, 150 and 300 or 6, 12, 24 and 48, were rinsed with distilled water to remove any extraneous particles and then transferred to rearing trays containing 1 l of distilled water. The first group of pupae were kept for 22 ± 2 h to produce the pupae-conditioned water (PCW). The second group of pupae were retained until all adults emerged (3 nights), and only the exuviae remained in the distilled water, after which the exuviae-conditioned water (XCW) was obtained. As controls, 1 l of distilled water was kept without pupae or pupal exuviae over the same time period under the same conditions. The conditioned water was then strained through nylon mesh and a folded filter paper (18.5 cm; Whatman International Ltd., Maidstone, England), and used immediately in subsequent assays.

### Multi-choice oviposition assay

To characterize the density-dependent effect of odours emanating from water conditioned with aquatic stages on oviposition site selection and egg laying by gravid *Ae. aegypti*, multi-choice oviposition assays were performed (Additional file 1: Fig. S1a). The conditioned water (40 ml of test or control) was added into the bottom section of an artificial oviposition site consisting of a blue plastic cup (250 ml; Duni, Malmö, Sweden), within which a second transparent cup (120 ml; ÖoB, Lund, Sweden), with six 2.5-mm-diameter perforations in the bottom, was placed in order to exclude input from sensory modalities other than olfaction (Additional file 1: Fig. S1b). A third cup (30 ml), containing distilled water and a filter paper, was placed inside the second cup (Additional file 1: Fig. S1b). The filter paper served as the oviposition substrate. Artificial oviposition sites containing the water conditioned with different densities of each aquatic stage and a distilled water control were placed in a BugDorm-1 cage. The treatments and control were randomly designated among the five artificial oviposition sites within the cage, one artificial oviposition site in each corner and one in the middle of the cage (Additional file 1: Fig. S1a). Five days after blood-feeding, an individual gravid mosquito (10–12 dpe) was released into each cage 2 h prior to scotophase, and provided ad libitum access to 10% sucrose. The placement of the sucrose dispensers alternated between the left and right sides of the cages, and had no effect on oviposition choice. The bioassays were kept under similar climate conditions for 48 h, as described above, after which the eggs laid in each artificial oviposition site were counted.

### Solid-phase microextraction headspace collections

Solid-phase microextraction (SPME) divinylbenzene/carboxen/polydimethylsiloxane Supelco StableFlex™ fibres (50/30 µm, 24 ga, 2 cm; Sigma-Aldrich, Stockholm, Sweden) were conditioned at 225 °C for 30 min using a gas chromatograph (GC; Agilent Technologies 6890, Santa Clara, CA, USA) prior to headspace collections. The glassware used for headspace collections was cleaned and placed at 250 °C for 8 h prior to use. Either the conspecific aquatic stage-conditioned water eliciting the highest oviposition response in the multi-choice assays (400 ml) or the control water (400 ml) was poured into 1 l glass bottles (VWR, Stockholm, Sweden). The water used for these odour collections was conditioned to match that of the conditioned water used in the behavioural experiments. Thereafter, sodium chloride (NaCl; ≥ 99%, Sigma-Aldrich), was dissolved into each to enhance the emission of volatiles following modified protocols of Lindh et al. [27] and Mozūraitis et al. [28]. Three concentrations of NaCl (150, 225 and 255 mg ml^−1^) were tested, from which it was determined that 255 mg ml^−1^ elicited the highest abundance of VOCs trapped on the SPME fibre from the conditioned water. The water was then incubated at room temperature for c. 5 min. The PCW was omitted due to the lack of a density-dependent oviposition response (Additional file 1: Fig. S2). The clean SPME fibre was introduced into a small hole (1.4 mm diameter) drilled in the polypropylene lid of the glass bottle containing the samples to collect the headspace for 17 h.

### Combined gas chromatography and electroantennographic detection

Antennal responses of 5-day post-blood-fed (10–12 dpe) *Ae. aegypti* to VOCs contained within the headspace collected on the SPME fibre of each of the aquatic stage-conditioned water were determined using combined gas chromatography (GC), flame ionization detection (FID) and electroantennographic detection (EAD) analyses. The GC (Agilent Technologies 6890) was equipped with an HP-5 column (30 m length × 0.25 mm inner diameter [i.d.] × 0.25 µm film thickness) and an effluent splitter between the column and the detectors. Hydrogen was used as the carrier gas at a linear flow rate of 45 cm s^−1^. The VOCs adsorbed onto the SPME fibres were injected into the GC in splitless mode for 1 min at 225 °C and thermally desorbed in the inlet. The GC oven temperature was programmed from 50 °C (hold for 1 min), increased at a rate of 8 °C min^−1^ to 275 °C (10 min hold). At the GC effluent splitter, nitrogen was added and the gas flow split between the FID and the EAD in a 1:1 volume four-way cross-splitter (Gerstel, Mülheim, Germany). The GC effluent moving towards the EAD passed through a Gerstel Olfactory Detector Port (ODP)-2 transfer line (Gerstel) that tracked the GC oven temperature before it was delivered into a glass tube (10 cm × 8 mm), where it was mixed with charcoal-filtered humidified air (1.5 l min^−1^). The antennal preparation was positioned 0.5 cm away from the outlet of the glass tube.

For the EAD analysis, a female mosquito was cold anesthetized prior to mounting the excised head on a reference electrode, which was inserted through the foramen. The cut, distal flagellomere of the antenna was connected to the recording electrode. Both electrodes were made from pulled glass microcapillaries, filled with Beadle–Ephrussi Ringer solution [29] and placed over chlorinated silver wires in the electrode holders. The recording electrode was attached to a pre-amplifier (1×), then to a high-impedance direct current (DC) amplifier interface (IDAC-2 Ockenfels Syntech GmbH, Buchenbach, Germany) and finally to a personal computer (PC) for visualization and storage. Three to five stable recordings were performed for the headspace collected from the water conditioned with each aquatic stage. The data were analysed using GC-EAD software (v.1.2.3, Ockenfels Syntech GmbH).

### Chemical analysis

The SPME samples from each of the aquatic stage-conditioned water and the corresponding controls were injected into a combined GC (6890, Agilent Technologies) and mass spectrometer (MS, 5975, Agilent Technologies) operated in the electron ionization mode at 70 eV. The GC–MS unit was equipped with the same type of fused silica capillary HP-5 column (60 m length × 0.25 mm i.d. × 0.25 µm film thickness) as the GC–EAD. Helium was used as the carrier gas at a linear flow rate of 34 cm s^−1^. The same temperature programme was used for both the GC–MS and the GC–EAD analyses. A total of three to five SPME samples, collected from the water conditioned with each of the aquatic stages and control, were injected into the GC–MS. All physiologically active VOCs, which were determined using GC–EAD analysis, were identified using linear retention times (Kovats index) and mass spectra in comparison with the National Institute of Standards and Technology (NIST) 17 library, and subsequently confirmed with authentic standards (Table 1). The relative abundance of the bioactive VOCs in each of the extracts was approximated by comparing the areas of each total ion chromatogram. The approximate relative abundance of each compound was then used to formulate the synthetic blends. The composition and ratio of, as well as the physiological response to, the compounds used in the synthetic blends were confirmed using GC–MS and GC–EAD, respectively. The purity of the commercial compounds was confirmed by injection on the GC–MS (Table 1).

**Table 1:** Physiologically bioactive volatile organic compounds identified from conspecific stage-conditioned water through GC–EAD and GC–MS analyses, and used for bioassays

Retention time	Retention indices	Compound	Purity (%)
6.56	839	2,4-Dimethylhept-1-ene	99.4
8.76	957	(*E*)-2-Heptenal	95.3
10.04	1023	4-Cyanocyclohexene	97.9
10.74	1059	(*E*)-2-Octenal	95
10.89	1072	2,6-Dimethyl-7-octen-2-ol	100
11.64	1105	Nonanal	99.4
12.50	1156	Camphor	99
12.70	1162	(*E*)-2-Nonenal	97.6
13.54	1207	Decanal	99.5
14.57	1264	(*E*)-2-Decenal	96.4
14.88	1281	Unknown	
16.93	1402	4-(2-Methylbutan-2-yl)phenol	99.8

### Dual-choice oviposition bioassay with synthetic blends

To determine the behavioural preference of gravid *Ae. aegypti* to the identified synthetic blends from the water conditioned with the different conspecific aquatic stages, a dose-dependent analysis was performed in a dual-choice oviposition assay. The artificial oviposition sites, containing either the treatment or the control, were placed in opposite corners of a BugDorm-1 cage, c. 8 cm from each cage wall. Each dilution of an individual synthetic blend (6 ml) was tested against a solvent control (6 ml hexane). Both the blend and the control were delivered from wick dispensers [30], constructed of a 12-ml vial (Genetec, Stockholm, Sweden) with a perforated lid (2-mm diameter hole) and a wick. The wick was made from Teflon tubing (75 mm length × 1.68 mm i.d. × 0.30 mm wall thickness), with a piece of unbleached cotton string inserted [30]. The wick dispensers allowed for the control of the release rate and ratio of the VOCs in the blend during the bioassay [30]. Each wick dispenser was then placed in a 250 ml glass wash bottle (VWR). Charcoal-filtered air was passed through the glass wash bottles, via Teflon tubing (6 mm outer diameter [o.d.]), using an air pump (model V-20; Guangdong Hailea Group Co., Ltd., Guangdong, China), into two 12-channel flow meters (Kytola Instruments, Muurame, Finland), in which the flow rate was adjusted to 0.1 l min^−1^. Teflon tubing connected the flow meters to the artificial oviposition sites (Additional file 1: Fig. S3) [31]. Individual 5-day post-blood-fed *Ae. aegypti* were introduced into each of the 12 BugDorm-1 cages containing the artificial oviposition sites, 2 h prior to scotophase. Females were offered ad libitum access to 10% sucrose during the bioassay (19 ± 1 h). Subsequently, the number of eggs laid in the treatment and the control was counted and oviposition choice indices were calculated: C/(C + T) and T/(T + C), in which C is the number of eggs laid in the control and T is the number of eggs laid in the treatment. Dual-choice assays with solvent-filled wick dispensers on both sides determined that there was no positional bias in the egg-laying choice of the gravid mosquitoes (Additional file 1: Fig. S4) [31]. Three or four replicates, each containing 12 mosquitoes, were performed.

### Statistical analysis

The Shapiro–Wilk test was performed to test for a normal distribution of eggs laid by each female, which determined that all the datasets failed the assumption of normality (JMP Pro version 16, SAS Institute Inc., Cary, NC, 1989–2021). The datasets for the multi-choice assays across different densities of each aquatic stage, and across the most preferred densities of a conspecific aquatic stage, were compared using the average total number of eggs laid per female by analysis of variance (ANOVA) followed by a Tukey post-hoc test. For the multi-choice assays, the artificial oviposition sites were rotated through each of the five positions, to minimize any location bias. Following the ECW and XCW experiments, the data were analysed using a general linear model (JMP Pro version 16), determining that there was no positional bias in the egg-laying response of gravid *Ae. aegypti* to these treatments, ECW (*χ*^2^ = 12.84, *df* = 8, *P* = 0.12) and XCW (*χ*^2^ = 9.71, *df* = 8, *P* = 0.29). For dual-choice assays, a binary logistic regression followed by odds ratio comparison was used to test for oviposition site preference, while, an ANOVA followed by a Tukey post-hoc test was used to assess egg stimulation in response to both treatments and controls (JMP Pro version 16). The egg-laying response was the dependent variable determined by the number of gravid females in the oviposition bioassay, and dose was the independent fixed effect.

## Results

### Water conditioned with aquatic stages affects oviposition

**Fig. 1:**
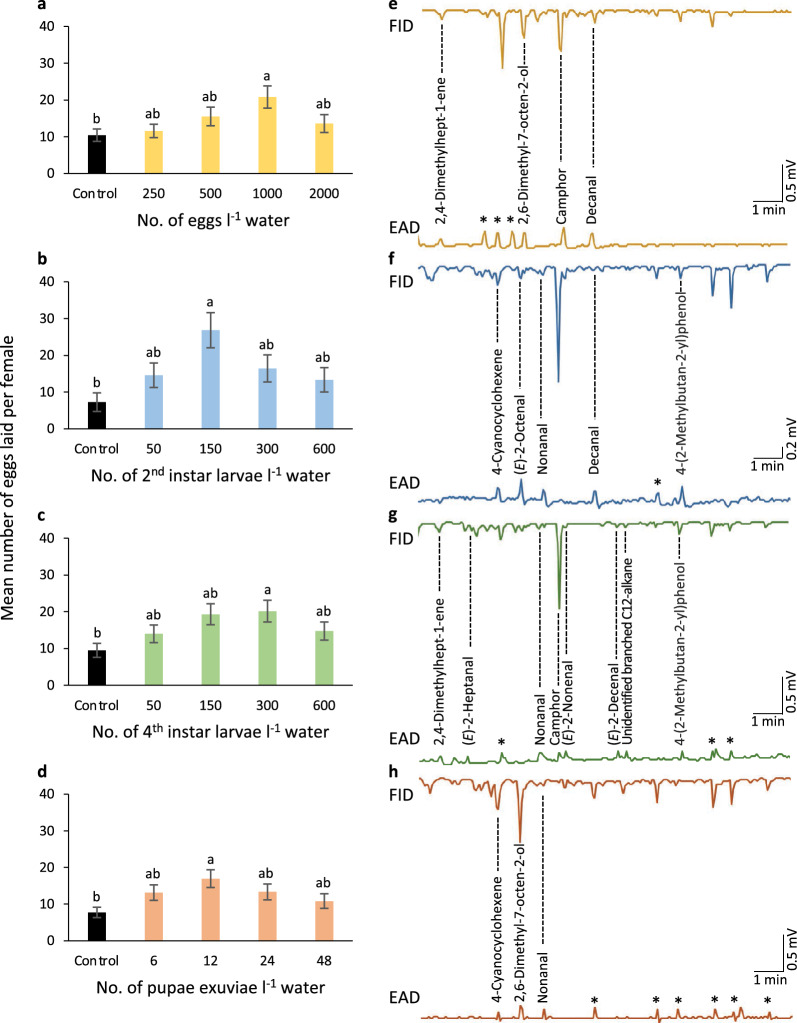
Behavioural and physiological responses of gravid *Aedes aegypti* to volatiles emanating from conspecific stage-conditioned water. The number of eggs laid by female mosquitoes in response to water conditioned with different densities of **a** eggs, **b** second-instar larvae, **c** fourth-instar larvae and **d** pupal exuviae. The different lowercase letters indicate significant differences (*P* < 0.05), as determined by an ANOVA followed by a Tukey post-hoc test. Error bars represent the standard error of the mean. In **e–h**, the combined gas chromatograph, flame ionization detection and electroantennographic detection (EAD) analyses demonstrate antennal responses of *Ae. aegypti* (mV) in response to bioactive volatile organic compounds in the headspace of conspecific immature-conditioned water eluting over time (min) from the gas chromatograph, and detected by the flame ionization detector (FID). Asterisks in the EAD traces represent compounds that were also present in the control SPME headspace

**Fig. 2:**
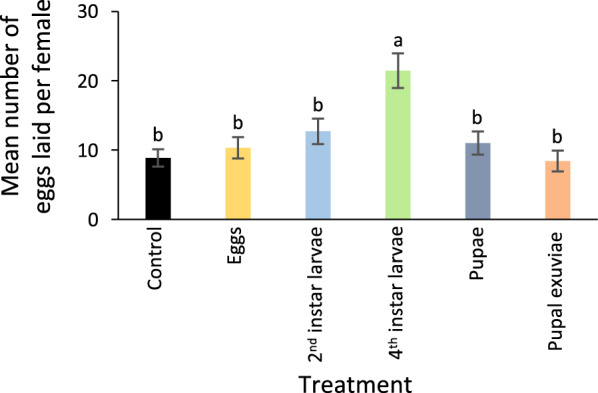
*Aedes aegypti* chooses to oviposit in response to volatiles from late instar-conditioned water. The different lowercase letters denote significant differences (*P* < 0.05), as determined by ANOVA followed by a Tukey post-hoc test. Error bars represent standard error of the mean

To assess the effect of water conditioned with different densities of aquatic stages of *Ae. aegypti* on the behavioural response of gravid mosquitoes, a series of multi-choice oviposition assays were conducted (Fig. 1a–d; Additional file 1: Figs. S1 and S2). Gravid mosquitoes demonstrated an overall density-dependent response to water conditioned with each aquatic stage compared to the control, as assessed using an ANOVA followed by a Tukey post-hoc test, for ECW (*F* = 2.72, *P* = 0.029); second-instar LCW (*F* = 3.89, *P* = 0.0042); fourth-instar LCW (*F* = 2.90, *P* = 0.021); and XCW (*F* = 2.76, *P* = 0.027); but not for PCW (*F* = 0.61, *P* = 0.66; Fig. 1a–d; Additional file 1: Fig. S2). In all experiments, gravid mosquitoes demonstrated a preference for conditioned water that had contained an intermediate density of the aquatic stages (Fig. 1a–d), with the exception of PCW, for which there was no preference for any density (Additional file 1: Fig. S2). When gravid mosquitoes were given the choice of the most preferred density of ECW, second-instar and fourth-instar LCW, PCW, and XCW in a subsequent multi-choice oviposition assay, individual gravid mosquitoes preferred to lay significantly more eggs in water conditioned with fourth-instar larvae (*F* = 7.46, *P* < 0.0001; Fig. 2).

### Bioactive compounds identified in water conditioned with aquatic stages

While combined GC–EAD and GC–MS analyses identified nine bioactive VOCs associated with the ECW extracts, only four (2,4-dimethylhept-1-ene, 2,6-dimethyl-7-octen-2-ol, camphor and decanal) were present in this treatment and not in the control, and thus are considered egg-associated compounds (Fig. 1e). Similarly, of the six and 11 bioactive VOCs from the second- and fourth-instar LCW, five (4-cyanocyclohexene, (*E*)-2-octenal, nonanal, decanal and 4-(2-methylbutan-2-yl)phenol) and eight (2,4-dimethylhept-1-ene, (*E*)-2-heptanal, nonanal, camphor, (*E*)-2-nonenal, (*E*)-2-decenal and 4-(2-methylbutan-2-yl)phenol, and an unidentified branched C12-alkane) were found in these treatments and not in their controls (Fig. 1f, g). From the XCW samples, nine VOCs elicited a response from the antennae, three of which (4-cyanocyclohexene, 2,6-dimethyl-7-octen-2-ol and nonanal) were present in this treatment and not in the associated control (Fig. 1h). While *n*-heneicosane, a previously identified putative oviposition pheromone component in *Ae. aegypti* larvae [32, 33] was specifically sought for in each of the SPME collections, none was identified in the GC–MS analysis.

### Synthetic odour blends elicit oviposition in *Ae. aegypti*

**Fig. 3:**
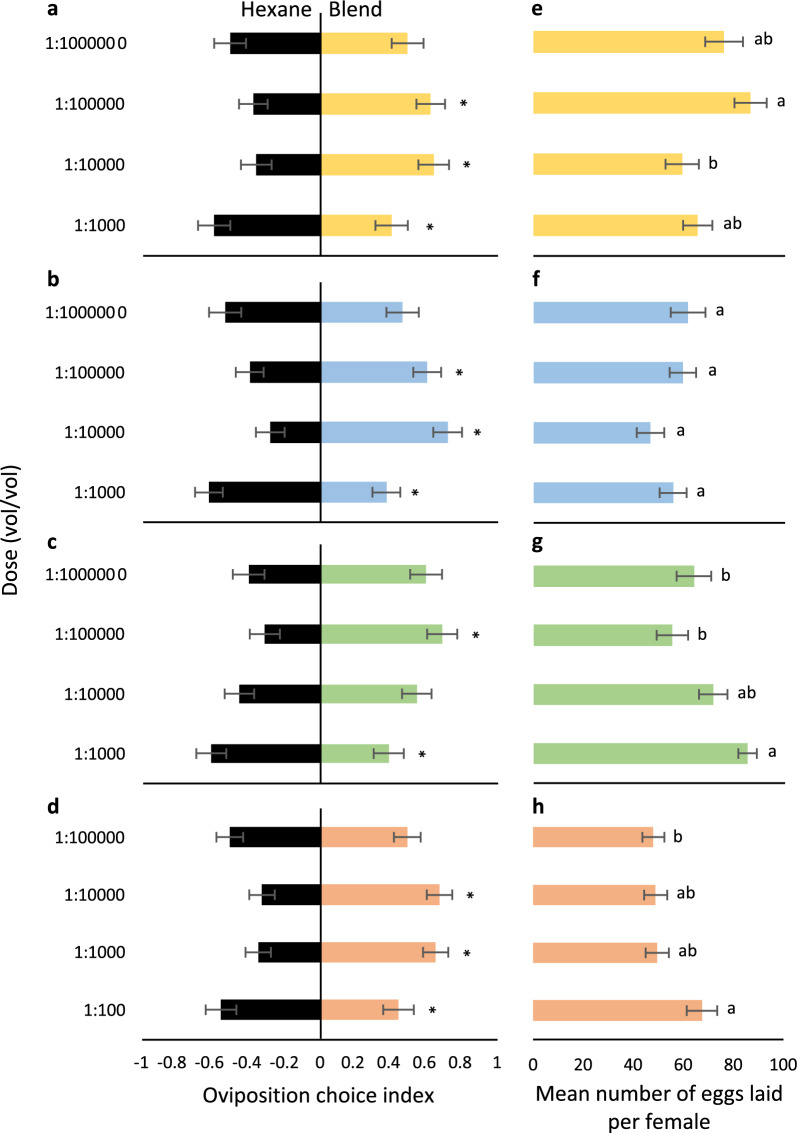
Synthetic odour blends of aquatic stage-conditioned water regulate oviposition choice and egg-laying in *Aedes aegypti*. The dose-dependent oviposition choice by gravid mosquitoes to individual synthetic odour blends identified as associated with water conditioned with **a** eggs, **b** second-instar larvae, **c** fourth-instar larvae and **d** pupal exuviae, in comparison with a solvent control (hexane). Asterisks indicate significant differences (*P* < 0.05), as determined by a nominal logistic general regression analysis followed by an odds ratio comparison. Gravid *Ae. aegypti* were differentially stimulated to lay eggs in response to these treatments (**e–h**). The different lowercase letters represent significant differences (*P* < 0.05), as determined by an ANOVA followed by a Tukey post-hoc test. Error bars represent the standard error of the mean. The sample size is greater than 30 for all comparisons

To assess whether synthetic odour blends, designed based on the bioactive VOCs identified to be associated with the aquatic stages of *Ae. aegypti*, elicit attraction and stimulation of oviposition, these were evaluated in a dual-choice assay and compared with a solvent control (hexane) (Additional file 1: Fig. S3). The synthetic blends were prepared to mimic the identified ratio of bioactive VOCs: eggs (2,4-dimethylhept-1-ene: 2,6-dimethyl-7-octen-2-ol: camphor: decanal, 6:1:17:1); second instar (4-cyanocyclohexene: (*E*)-2-octenal: nonanal: decanal: 4-(2-methylbutan-2-yl)phenol, 1:7:9:5:17); fourth instar (2,4-dimethylhept-1-ene: (*E*)-2-heptanal: nonanal: camphor: (*E*)-2-nonenal: (*E*)-2-decenal: 4-(2-methylbutan-2-yl)phenol, 11:9:3:48:1:9:20); and pupal exuviae (4-cyanocyclohexene: 2,6-dimethyl-7-octen-2-ol: nonanal, 1:6:4), respectively. The four blends were diluted in hexane and assayed at different doses against a solvent control (hexane) (Fig. 3a–d). Gravid *Ae. aegypti* were attracted to oviposit in a dose-dependent manner in response to each of the four synthetic blends: eggs (*χ*^2^ = 12.09, *df* = 3, *P* = 0.0071); second instars (*χ*^2^ = 15.22, *df* = 3, *P* = 0.0016); fourth instars (*χ*^2^ = 6.79, *df* = 3, *P* = 0.079); and pupal exuviae (*χ*^2^ = 11.11, *df* = 3, *P* = 0.011) (Fig. 3a–d). Moreover, the synthetic blends significantly stimulated the gravid *Ae. aegypti* to lay eggs dose-dependently, except in response to that of the second instars: eggs (*F* = 3.39, *P* = 0.020); second instar (*F* = 1.22, *P* = 0.30); fourth instar (*F* = 4.90, *P* = 0.0030); and pupae exuviae (*F* = 3.13, *P* = 0.027) (Fig. 3e–h). While a higher overall number of eggs were laid in response to the synthetic odour blends based on the VOCs identified associated with eggs and fourth-instar LCW (Fig. 3e, g), the eggs were laid differentially, with those laid in response to the egg-based odour blend being predominantly placed in the treatment site, and those laid in response to the fourth-instar-based odour blend being predominantly laid in the control site (Fig. 3e, g).

## Discussion

Volatiles associated with conspecific aquatic stages differentially attract mosquitoes to oviposition sites and stimulate egg laying [this study, 11, 12, 15, 17, 18, 20, 24, 34–40]. In this study, gravid *Ae. aegypti* were preferentially attracted to oviposit in response to the VOCs emanating from water conditioned with late-stage larvae [11, 20, 22], which likely signals a productive breeding site and reduced competition for resources between the existing, soon to pupate, larvae and the new generation [6, 20, 41, 42]. Furthermore, the density of the conspecific aquatic stages affected oviposition site choice, with gravid mosquitoes laying fewer eggs, and even avoiding ovipositing, on the treated sites when the odours of these sites indicated high densities of conspecific competitors [this study, 12, 15, 17, 18, 24, 25, 41, 43]. Both the aquatic stage and odour release rate significantly affected the manner in which females were stimulated to oviposit in either the treated or controlled sites. Our findings indicate that gravid mosquitoes rely on the detection of aquatic stage-specific VOC blends for the identification and discrimination among potential oviposition sites to provide a reliable signal of the suitability of a potential breeding site for their offspring [24, 39]. This differential preference for a specific conspecific aquatic stage, and its volatiles, may have a direct effect on the population dynamics of *Ae. aegypti*, and provide a potential route by which to manipulate vector behaviour.

The presence of conspecific aquatic stages in a breeding site, currently or in the recent past, influences and mediates the oviposition site selection and egg-laying decision of gravid mosquitoes [this study, 11, 12, 15, 17, 18, 20, 24, 34–40, 44]. The behavioural responses of gravid mosquitoes to these sites are species- and taxon-specific [34, 36, 38, 41, 44]. For example, gravid *Ae. aegypti* preferentially oviposit in water conditioned with late-stage larvae [this study, 20], whereas *Anopheles coluzzii* preferentially lay eggs in breeding sites containing first-instar larvae [15]. This indicates that the odour profile of breeding sites changes depending on the developmental stage of conspecifics, as demonstrated in this study for *Ae. aegypti*, and that of Schoelitsz et al. [39] for *An. coluzzii*.

The ecological rationale for the observed taxon-specific strategies of gravid mosquitoes to use cues from particular conspecific aquatic stages as indicators of the suitability of a potential breeding site for their offspring is based on species-related differences in the ability of their offspring to withstand the changing biotic (e.g., food resources, competition) and abiotic (e.g., water availability, dissolved oxygen, salinity) conditions in the breeding sites [2, 5, 19, 20, 34, 36, 38, 41, 44–46]. *Aedes aegypti* selects breeding sites which contain/contained late-stage larvae for oviposition, which may at first seem counter-intuitive, as by the time that larvae reach the fourth instar there is the risk that these will have consumed the bulk of the resources in the restricted, local environment, and may compete with newly hatched larvae for the limited resources contained within [20, 47]. However, late-stage larvae will soon transition to the non-feeding pupal stage, thereby reducing the risk of conspecific competition with newly hatched larvae, reflecting the oviposition preference of *Ae. aegypti* under natural and laboratory conditions [this study, 20]. The lack of attraction of the gravid mosquitoes to the conditioned water associated with the pupal stage, as the pupae do not produce the volatile signals attracting the gravid females [this study], reflects the inability of the pupae to excrete and thereby to affect the microbiota and uric acid in the environment [48, 49]. Upon adult emergence, on the other hand, the meconium harboured within the pupa is egested, releasing the gut microbiota and other urate-based wastes into the breeding site [49]. Moreover, infested breeding containers typically contain single cohorts of aquatic stages developing in synchrony [50], demonstrating that gravid mosquitoes only recolonize breeding sites in the presence of conspecific eggs, to which gravid mosquitoes are stimulated to lay larger clutches of eggs, or late aquatic stages [this study, 20], to further reduce competition for their offspring. Indirect effects related to the development of the aquatic stages of *Ae. aegypti* may also contribute to oviposition site preference. Breeding sites showing signs of high levels of microbial growth, specifically microbes inoculated during oviposition [51–54], and associated detritus [55–57] that have accumulated during conspecific larval development, provide a rich, abundant larval food source, which reduces the potential competition between established and newly hatched larvae [58]. Gravid mosquitoes ovipositing in temporary waters, such as *Ae. aegypti*, may also indirectly use abiotic factors to assess potential oviposition sites, as cannibalism increases with the increasing numbers of interactions between stages as density increases due to spatial limitation, rather than due to food restriction [14, 59].

Gravid mosquitoes respond in a density-dependent manner to water that contains, or has contained, conspecific aquatic stages [this study, 12, 15, 17, 18, 24, 60]. In our study, gravid *Ae. aegypti* laid fewer eggs in response to water conditioned with high densities of conspecific eggs, larvae and pupal exuviae, supporting the hypothesis that laying the majority of eggs in water with low densities of conspecific aquatic stages will enhance progeny growth, development and the probability of survival, as a result of reduced competition [23, 25, 61]. In contrast, high larval densities generate competition for food resources, which affects the hatching rate and larval development, as well as adult size and survival rate [23, 25, 61, 62]. While in other mosquito species, larvae that are overcrowded, and/or starved, emit deterrent VOCs, which negatively affect the oviposition behavioural response [15, 24, 43], such VOCs have yet to be demonstrated for *Ae. aegypti*. Together, these density-related factors affect the fitness of gravid mosquitoes, which in turn affects the vectorial capacity [61, 63–65]. The assessment of the factors associated with the stage and density of immature conspecifics by gravid mosquitoes can be in large part attributed to the quality and quantity of the VOCs emanating from the potential breeding sites.

Available data indicate that mosquito taxa use distinct blends of VOCs to mediate oviposition site selection and egg-laying behaviour in a species-dependent manner [this study, 11, 31]. While mosquitoes mainly respond to species-specific VOCs, gravid females make use of signature VOCs from breeding sites, particularly select straight-chain aldehydes, monoterpenes, and straight-chain fatty acids and esters, that are commonly detected across mosquito taxa [this study, 11 and references therein, 34, 35, 66–68]. The straight-chain aldehydes [(*E*)-2-heptanal, (*E*)-2-octenal, nonanal, (*E*)-2-nonenal, decanal, (*E*)-2-decenal] and monoterpenes (2,6-dimethyl-7-octen-2-ol, camphor) identified in this study have previously been demonstrated to be emitted by multiple resources used by mosquitoes to locate floral nectar [69–71], host [71, 72] and oviposition site sources [11, 73], reflecting chemical parsimony, suggesting that mosquitoes are under a strong adaptative pressure to make use of the same VOCs as signals to identify various resources [74]. In addition, these and other parsimonious VOCs, detected and used by gravid mosquitoes to locate species-dependent oviposition sites [6, 11], emanate from diverse resources, including conspecific stages [11], food resources [6, 75, 76], fermenting vegetation [77] and living vegetation associated with breeding sites [78–81]. For example, nonanal, decanal and camphor have been identified from several of the conspecific developmental stages of *Ae. aegypti* [this study, 45], but also from infusions of vegetation and grass pollen used by *Culex quinquefasciatus* and *Anopheles arabiensis* larvae as food sources, respectively [75–77, 82]. In addition, (*E*)-2-decenal has been reported from chicken faeces, which attracts *Culex* mosquitoes to preferred oviposition sites [73, 83]. Moreover, straight-chain fatty acids and esters associated with the aquatic stages of mosquitoes appear to be detected across the culicines, and play a role in oviposition site selection [34, 35, 66–68, 84]. While several straight-chain fatty acids and esters have been previously identified from the conspecific aquatic stages of *Ae. aegypti*, none were identified in this study. Moreover, the putative larval pheromone component, *n*-heneicosane [32, 33], was not identified from the larvae-conditioned water in this study, possibly due to the mostly insoluble nature of alkanes in water. The other compounds identified in this study are structurally diverse and have not previously been associated with oviposition sites. Evidence strongly suggests that gravid *Ae. aegypti* do not detect these VOCs singly, but rather as blends, or chemical codes, which enable them to discriminate among potential sites to lay their eggs.

The combined chemical and electrophysiological analyses of the stage-specific conditioned water extracts demonstrated partially overlapping, yet distinct, VOC blends that provide the basis for gravid *Ae. aegypti* to discriminate and select among potential breeding sites. In previous studies, single VOCs, identified from the chemical analyses of breeding sites, were tested in behavioural assays without prior experiments supporting their physiological activity. Here, we present a workflow that demonstrates the importance of combinatorial coding of VOCs by mosquitoes for oviposition site selection. This coding strategy is not restricted to conspecific signalling, but has also been demonstrated in *An. arabiensis* in relation to oviposition site selection among vegetative resources [75, 76, 79, 85]. An increased understanding of the chemical codes underlying oviposition site selection may lead to the development of novel odour lures used for vector control and surveillance [11, 73].

## Conclusions

Gravid *Ae. aegypti* use conspecific stage- and density-dependent cues to identify potential oviposition sites. The volatile profile of oviposition sites conditioned with conspecific aquatic stages changes throughout developmental time, in terms of the quality of the VOCs emitted, providing gravid mosquitoes a means by which to discriminate among oviposition sites, as well as cues for egg stimulation. The identified stage-specific synthetic odour blends regulating oviposition site selection in *Ae. aegypti* may be good candidates for the development of lures to be used in integrated vector control programmes. Future research will optimize and formulate synthetic lures, and subsequently investigate the combining of select blends with, e.g., the In2Care mosquito traps [86], to assess whether these will improve the efficacy of attract-and-kill mosquito control devices.

### Supplementary Information

**Additional file 1: Figure S1.** Multi-choice assay used to assess oviposition preference of *Aedes aegypti* to conspecific-conditioned aquatic stage water. **a.** The placement of the artificial oviposition sites (triple cups) within a BugDorm-1 cage. **b.** The construction of the triple cups, allowing olfactory cues, but no other sensory stimuli, to perfuse the assay. **Figure S2.** Oviposition site selection by gravid *Aedes aegypti* in response to pupae-conditioned water. The lowercase letters indicate no significant differences (*P* > 0.05), as determined by an ANOVA followed by a Tukey post-hoc test. Errors bars represent the standard error of the mean. **Figure S3.** Dual-choice oviposition assay used to evaluate choice and egg-laying of *Aedes aegypti* to synthetic blends. **Figure S4.** Oviposition choice of gravid *Aedes aegypti* to solvent controls (hexane) in a dual-choice assay. Gravid *Ae. aegypti* demonstrated no behavioural preference for either side of the oviposition assay (ANOVA followed by a Tukey post-hoc test). Error bars represent the standard error of the mean.

## Data Availability

All data generated or analysed during this study are included in this published article and its supplementary information files.

## Abbreviations

EAD: Electroantennographic detection
ECW: Egg-conditioned water
FID: Flame ionization detector
GC–MS: Gas chromatography–mass spectrophotometry
LCW: Larvae-conditioned water
PCW: Pupae-conditioned water
SPME: Solid-phase microextraction
VOCs: Volatile organic compounds
XCW: Exuviae-conditioned water
