# Supplementary figures and images for: Odour-mediated oviposition site selection in Aedes aegypti depends on aquatic stage and density
Source: Parasit Vectors. 2023 Aug 4;16:264. doi: 10.1186/s13071-023-05867-1 (PMC10403918; doi:10.1186/s13071-023-05867-1)

Figure S1

**a**

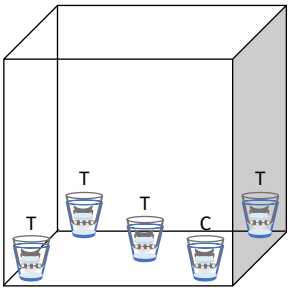

**b**

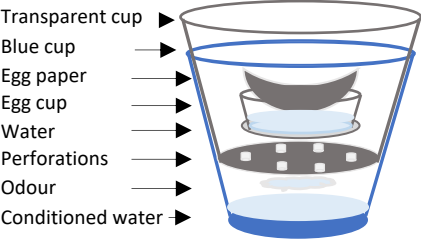

Figure S2

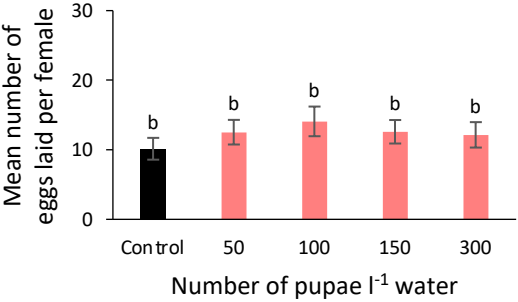

Figure S3

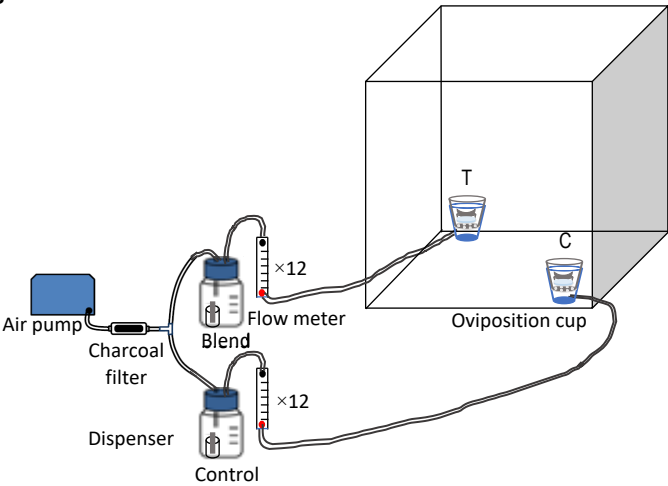

Figure S4

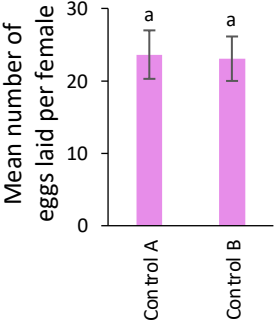

Supplement: Supplementary file 1 — Additional file 1: Figure S1. Multi-choice assay used to assess oviposition preference of Aedes aegypti to conspecific-conditioned aquatic stage water. a. The placement of the artificial oviposition sites (triple cups) within a BugDorm-1 cage. b. The construction of the triple cups, allowing olfactory cues, but no other sensory stimuli, to perfuse the assay. Figure S2. Oviposition site selection by gravid Aedes aegypti in response to pupae-conditioned water. The lowercase letters indicate no significant differences (P > 0.05), as determined by an ANOVA followed by a Tukey post-hoc test. Errors bars represent the standard error of the mean. Figure S3. Dual-choice oviposition assay used to evaluate choice and egg-laying of Aedes aegypti to synthetic blends. Figure S4. Oviposition choice of gravid Aedes aegypti to solvent controls (hexane) in a dual-choice assay. Gravid Ae. aegypti demonstrated no behavioural preference for either side of the oviposition assay (ANOVA followed by a Tukey post-hoc test). Error bars represent the standard error of the mean. [file 13071_2023_5867_MOESM1_ESM.pdf]
